# Supplementary material for: Protein accumulation in the endoplasmic reticulum as a non-equilibrium phase transition
Source: Nat Commun. 2014 Apr 11;5:3620. doi: 10.1038/ncomms4620 (PMC4048836; doi:10.1038/ncomms4620)
Supplement: Supplementary Information — Supplementary Figures 1-5, Supplementary Table 1, Supplementary Methods and Supplementary References [file ncomms4620-s1.pdf]

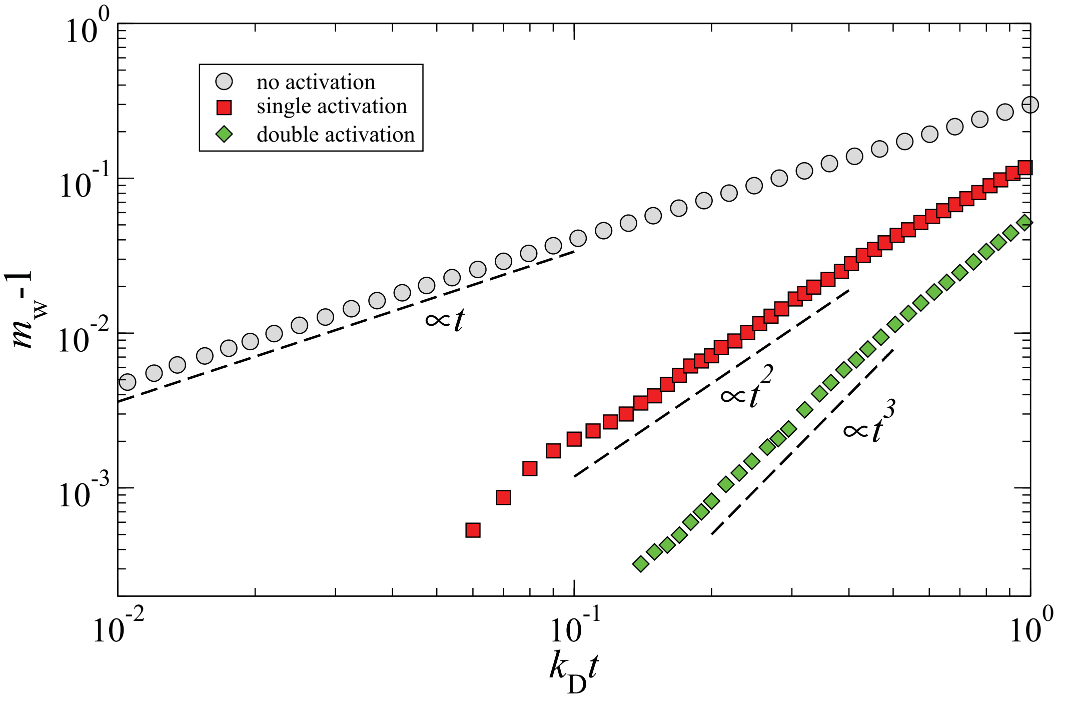

Supplementary Figure 1: **Role of monomer activation for polymerization**. Results of the initial growth of the polymer weighted mass in the three dimensional model for different activation rules: (i) No activation necessary to form dimers. (ii) It is enough that one of the two monomers is active in order to form a dimer. (iii) Both monomer must be active to form a dimer.

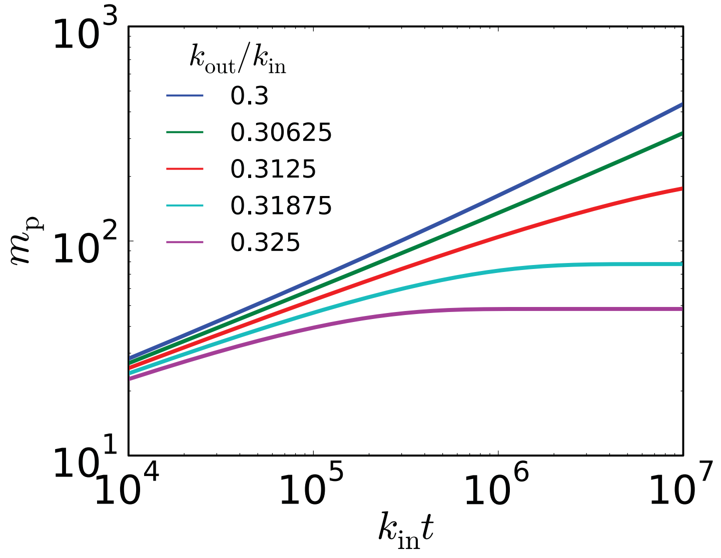

Supplementary Figure 2: **Transition between growing and stationary phases as $k_{\rm out}$ is increased**. The model is given by Eq. (19), and here parameters $k_{\rm in} = 1$, $k_{\rm p} = 1$, $k_{\rm f} = 1$ are used, with $f(i) = i^{-3}$ and cutoff $c = 40$. We plot against time rescaled by monomer production rate, $k_{\rm in}t$. In the growing (small $k_{\rm out}$) phase, the mean polymer size grows indefinitely as a power law t^β with $\beta \to 1/2$ as $k_{\rm out} \to 0$.

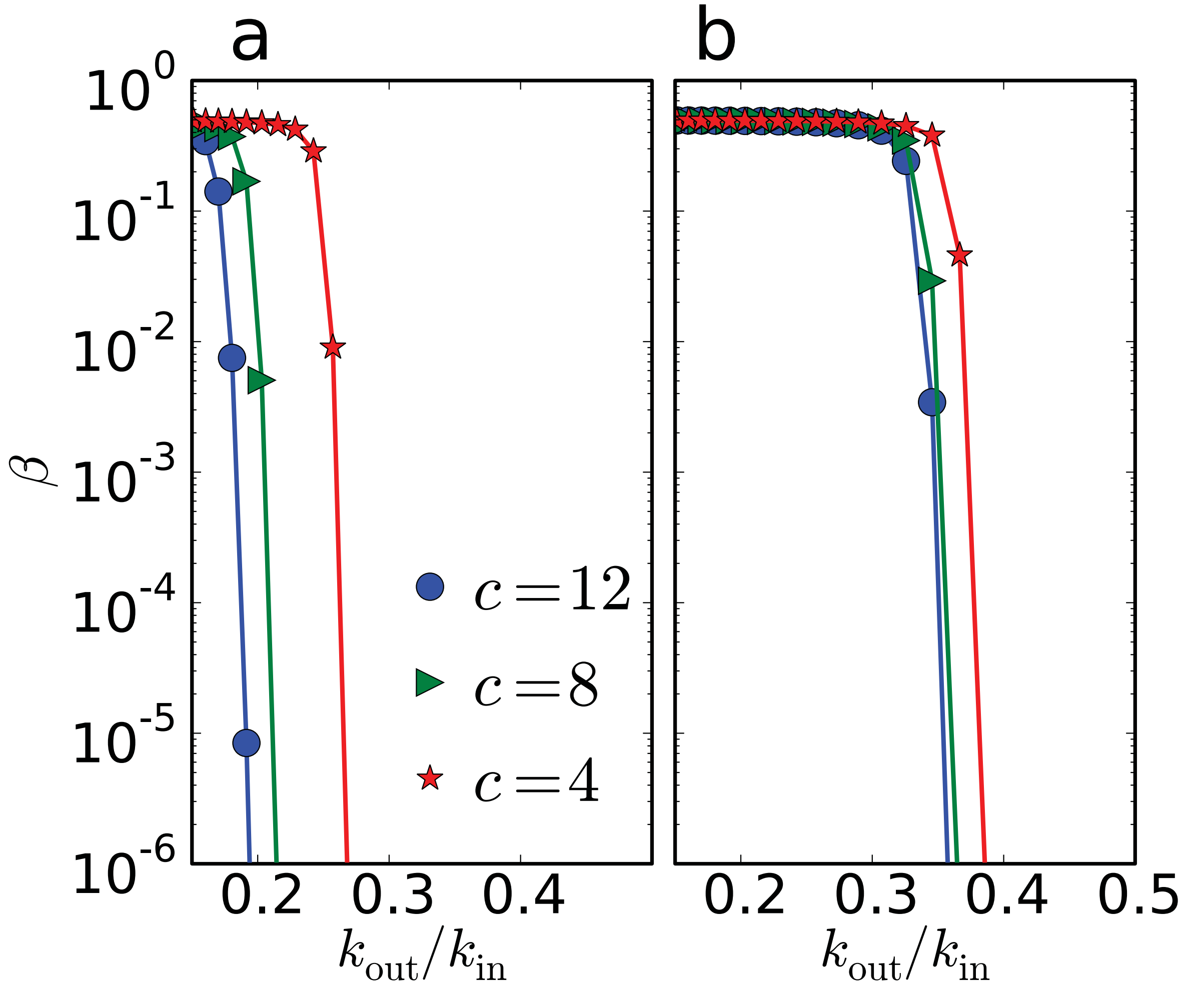

Supplementary Figure 3: **Role of the cutoff in the degradation rate**. The effective exponent, β, obtained from a power law fit of the long-time growth in mean polymer size, as a function of rate constant k_{out}, for fixed $k_{\text{in}} = k_{\text{p}} = k_{\text{f}} = 1$ and various cutoffs c for polymer degradation. In (a), the size dependence of the rate of polymer degradation is $f(i) = i^{-2}$ and as $c \to \infty$, the transition moves to $k_{\text{out}} = 0$; in (b) the rate is $f(i) = i^{-3}$ and the critical value of k_{out} remains finite.

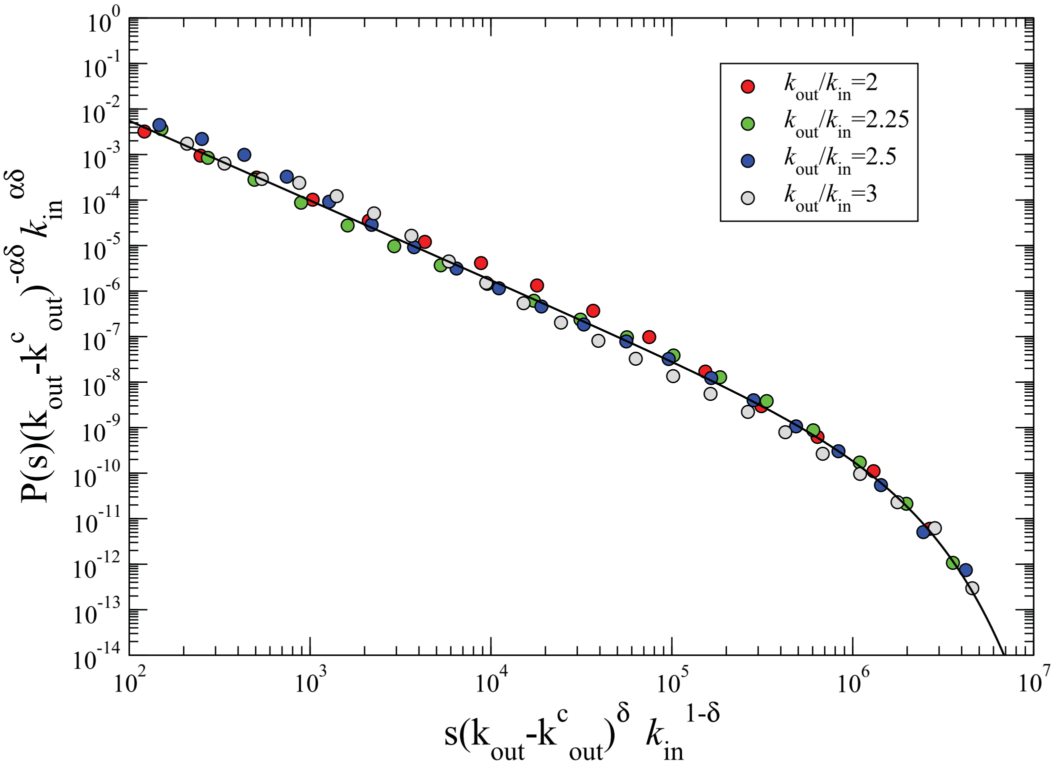

Supplementary Figure 4: **Avalanche size distributions**. a) The distribution of burst size (the area under a pulse) follows a power law distribution up to a characteristic cutoff that increases as the transition is approached. Above the transition, we observe large events that should be limited by system size. b) Different distributions can be collapsed onto a universal scaling function.

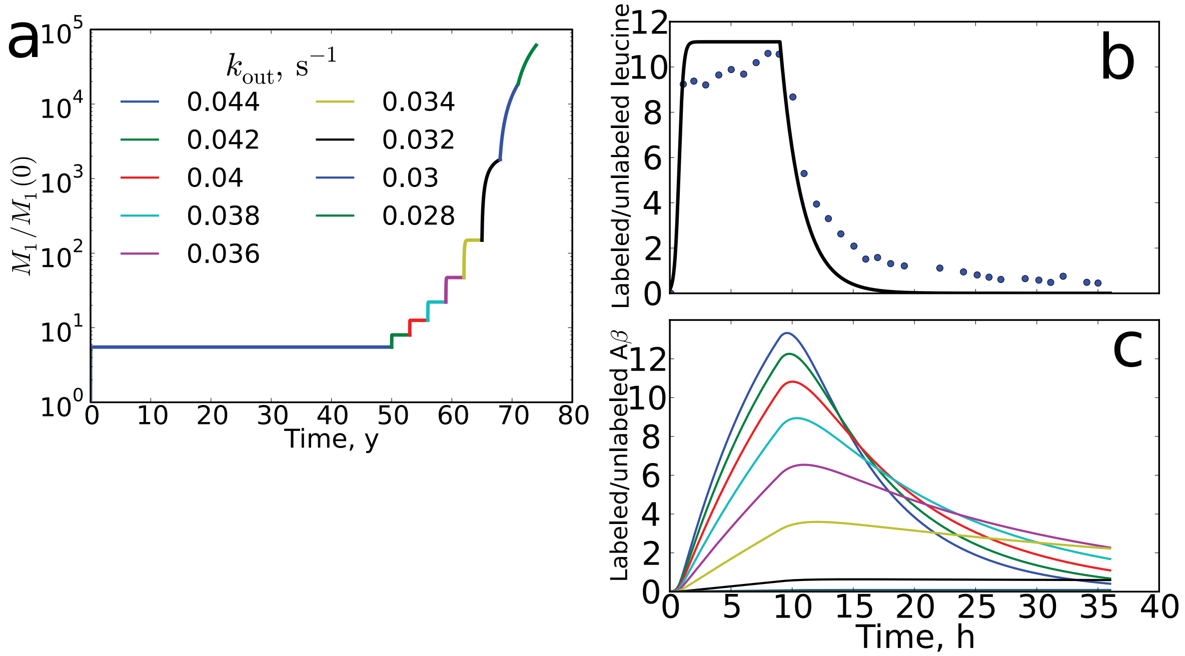

Supplementary Figure 5: **Effect of protein degradation rate k_{out} on amyloid-β synthesis and clearance**. (a) Evolution of total mass in the system as k_{out} is slowly reduced from values in the stationary phase to values in the growing phase. Time is measured in years. (b) Time course of labeled/unlabeled ratio for leucine (and hence monomers) in simulations. (c) Labeled/unlabeled mass ratios in the brain for labeling tests performed for each k_{out} value given in (a).

Parameter	Value
Production rate, k_{in}	5×10^{-3} s^{-1}
Degradation rate, k_{out}	0.044 s^{-1}
Aggregation rate*, k_{p}	5×10^{-3} s^{-1}
Fragmentation rate†, k_{f}	2×10^{-4} s^{-1}
CSF mixing rate‡, v	0.1667 h^{-1}
$\ell(t)$ maximum value§	0.1

Supplementary Table I: Parameter values used for the initial leucine labeling test reported in Fig. 5.

* Based on a rate of 5×10^{-3} M^{-1} s^{-1} with a concentration of 1 μM, consistent with *in vitro* values given in Fig. 3 of Ref. [1].

† This is larger than *in vitro* values reported in, e.g, Ref. [1], however factors such as mechanical agitation, temperature and the activity of molecular chaperones [2, 3] can all increase fragmentation *in vivo*.

‡ Based on CSF volume of 120 ml and production of 20 ml h^{-1} [4].

§ Based on fitting the CSF leucine labeled fraction in Fig. 3b of Ref. [5].

Supplementary Methods

Multi-parameter curve fitting

We fit the polymerization kinetics reported in Fig. 2a by a crossover function

$$m_{\mathrm{w}} - 1 = A \frac{t^2}{(1 + (Bt\rho^{\gamma})^{\frac{2-\beta}{\kappa}})^{\kappa}}, \tag{1}$$

crossing over between t^2 and t^{β} at a characteristic time $\tau = 1/(B\rho^{\gamma})$. The shape parameter κ tunes the sharpness of the crossover. We fit simultaneously with Eq. (1) all the curves obtained at different densities using the pyfitting python routine (https://github.com/gdurin/pyFitting). The best fit parameters are $\gamma = 0.149 \pm 0.002$, $\kappa = 2.00 \pm 0.03$, $\beta = 0.516 \pm 0.004$, $A = 12.6 \pm 0.1$, $B = 0.65 \pm 0.01$, with a relative $\chi^2 = 0.001$. The same method is used to fit the distributions reported in Fig. 4b.

Details on three dimensional model

In the model, individual proteins are modelled as monomers sitting on a three dimensional square lattice. Monomers diffuse with rate $k_{\rm D}$ and attach to neighboring monomers or polymer endpoints with rate $k_{\rm H}$. Polymers move collectively by reptation with a length-dependent rate $k_{\rm R}/i^2$, where i is the number of monomers in the polymer (see Ref. [6] p. 89), and locally by end rotations, with rate $k_{\rm E}$ and kink moves with rate $k_{\rm K}$ (for a review of lattice polymer models see [6]). A polymer can attach to another polymer with rate $k_{\rm H}$ if their endpoints meet, and can fragment by breaking an internal bond with rate $k_{\rm f}$ (Fig. 1c). Inspired by experimental results on neuroserpin polymerization [7], we allow for polymerization after at least one of the monomers has been activated with rate $k_{\rm A}$. Active monomers can also become latent with rate $k_{\rm L}$ and after that they do not aggregate (Fig. 1d).

We consider two types of boundary and initial conditions for the polymerization kinetics. (i) To simulate experiments in vitro, we start with a constant number of inactive monomers in a cubic system of size $L = 60L_0$, where L_0 is the typical monomer diameter, with periodic boundary conditions in all directions. (ii) To simulate polymerization in the ER, we consider a system of size $(L \times L \times H)$ with $L = 100L_0$ and $H \ll L$ with periodic boundary conditions along x and y and closed boundary conditions along z. Most simulations are performed with $H = 25L_0$. Monomers enter the system from the closed boundaries with rate $k_{\rm in}$ and polymers can exit from the system with rate $k_{\rm out}/i^3$. We have checked that other functional dependencies of the exit rate on the number of monomers in the polymer yield similar results. We perform numerical simulations using Gillespie Montecarlo algorithm [8]. In case (i) we measure time in units of $1/k_{\rm D}$, setting $k_{\rm H} = k_{\rm E} = k_{\rm R} = k_{\rm K} = k_{\rm A} = k_{\rm D}$ and we vary ρ, $k_{\rm L}$ and $k_{\rm f}$. In case (ii) we measure time in units of $1/k_{\rm in}$ and set $k_{\rm H} = k_{\rm E} = k_{\rm R} = k_{\rm K} = k_{\rm A} = k_{\rm in}$, $k_{\rm L} = 0$, $k_{\rm D} = 10^2 k_{\rm in}$, $k_{\rm f} = 10^{-3} k_{\rm in}$ and vary $k_{\rm out}$.

Solution of the mean-field model

In mean field models, the evolution of a system of interacting polymers is given by an infinite set of coupled nonlinear first order differential equations that describe how the populations n_i of i-mers evolve. Such sets of equations have been studied extensively since

Smoluchowski's pioneering work in 1917 [1, 9–15]. In the context of amyloids in particular, a major focus of mean field modeling has been the question of how polymers are initially nucleated [1, 16–21]. In those works, the standard approach is to maintain a constant total mass density in the system, that is $M_1 = \sum_i in_i = \text{const}$, however in order to model protein accumulation *in vivo*, where monomers are created from amino acids and polymers are removed by protein degradation, a model with mass flux is required.

Models with conserved mass

Before discussing the effect of monomer production and polymer degradation, we first recap several constant-mass models that have appeared previously in the literature. Such models are appropriate to describe *in vitro* experiments in which the total mass is conserved. We show here that a simple model is sufficient for our purposes.

In order to illustrate the approach taken, we demonstrate the solution of the simplest possible model, that of Smoluchowski [9, 22], in which polymer aggregation is the only process that occurs. Such as system is described by equations for the concentration n_i of polymers of size i, $i \geq 1$:

$$\dot{n}_i = \frac{1}{2} k_\mathrm{p} \sum_{j=1}^{i-1} n_j n_{i-j} - k_\mathrm{p} n_i \sum_{j=1}^{\infty} n_j. \tag{2}$$

The first term on the right hand side gives the increase in n_i due to the aggregation of two smaller polymers, and the second term gives the decrease in n_i due to a polymer of size i attaching to another polymer. In all that follows, n_i is defined by the number of polymers of size i in the system, normalized by the initial total mass, so that $\sum_i in_i = 1$. (Elsewhere in the literature, other conventions are sometimes used.) The rate constant, k_p, is $k_\mathrm{p} = DR\mu/V$, where D is the diffusion constant, R is the 'radius of influence' of a polymer, μ is the initial total mass (in Mol), V is the system volume, and the product DR is assumed independent of polymer size [22].

The moments of the mass distribution of the system described by Eq. (2) can be found by summation of all terms. Because mass is conserved, $M_1 = \sum_i in_i = 1$ always. The zeroth

moment, $M_0 = \sum_i n_i$ is the total number of polymers, and is given by

$$\begin{aligned} \dot{M}_0 &= \sum_{i=1}^{\infty} \dot{n}_i, \\ &= \frac{1}{2} k_{\mathrm{p}} \sum_{i=1}^{\infty} \sum_{j=1}^{i-1} n_j n_{i-j} - k_{\mathrm{p}} \sum_{i=1}^{\infty} n_i \sum_{j=1}^{\infty} n_j, \\ &= -\frac{1}{2} k_{\mathrm{p}} M_0^2, \end{aligned} \tag{3}$$

where the final step results from rearranging the summation. The resulting equation for M_0 can be solved analytically, to yield

$$M_0(t) = \frac{1}{1 + \frac{k_{\mathrm{p}}}{2} t}. \tag{4}$$

In the same way, we can also obtain the second moment, $M_2 = \sum_i i^2 n_i$:

$$\begin{aligned} \dot{M}_2 &= \sum_{i=1}^{\infty} i^2 \dot{n}_i, \\ &= \frac{1}{2} k_{\mathrm{p}} \sum_{i=1}^{\infty} i^2 \sum_{j=1}^{i-1} n_j n_{i-j} - k_{\mathrm{p}} \sum_{i=1}^{\infty} i^2 n_i \sum_{j=1}^{\infty} n_j, \\ &= k_{\mathrm{p}} M_1^2. \end{aligned} \tag{5}$$

In other words, the second moment grows linearly in time.

While the aggregation-only model is straightforward to solve, it lacks certain key features that are required to model protein aggregation dynamics in the cell, where polymers can fragment as well as aggregate. In particular, the aggregation model yields continuous linear growth of the mean polymer size always, which is unrealistic. We therefore focus on a model that incorporates fragmentation, based on that of Blatz and Tobolsky in 1945 [10], in which the evolution of the population n_i of polymers of size i, $i \geq 1$, is given by

$$\dot{n}_i = \frac{1}{2} k_{\mathrm{p}} \sum_{j=1}^{i-1} n_j n_{i-j} - k_{\mathrm{p}} n_i \sum_{j=1}^{\infty} n_j - k_{\mathrm{f}} n_i (i-1) + 2 k_{\mathrm{f}} \sum_{j=i+1}^{\infty} n_j. \tag{6}$$

The terms are:

- $\frac{1}{2} k_{\mathrm{p}} \sum_{j=1}^{i-1} n_j n_{i-j}$ gives the increase in n_i due to two smaller -mers aggregating;
- $-k_{\mathrm{p}} n_i$ gives the decrease in n_i due to an i-mer aggregating to another -mer;

- $-k_\mathrm{f} n_i (i-1)$ gives the decrease in n_i due to an i-mer fragmenting into two smaller -mers, with the factor $(i-1)$ accounting for the number of locations a breakage can occur; and

- $2k_\mathrm{f} \sum\limits_{j=i+1}^{\infty} n_j$ gives the increase in n_i due to a larger -mer fragmenting.

The fragmentation rate, k_f is independent of the total polymer mass and system volume.

As shown by Blatz and Tobolsky [10], this system can be solved analytically to reveal the roles of k_p and k_f. This is done by writing equations for $M_0 = \sum_{i \geq 1} n_i$ and $M_1 = \sum_{i \geq 1} i n_i$, which yields

$$M_1 = 1, \tag{7}$$

$$M_0 = 1 - p, \tag{8}$$

$$n_1 = (1-p)^2, \tag{9}$$

where

$$p = \big(K + \sqrt{K^2 - 1} \coth(\frac{k_\mathrm{p} t}{2} \sqrt{K^2 - 1})\big) \tag{10}$$

and

$$K = 1 + k_\mathrm{f}/k_\mathrm{p}. \tag{11}$$

(Blatz and Tobolsky use notation where k_b is the 'backward' rate and corresponds to our k_f, and k_f is the 'forward' rate and corresponds to our k_p.)

The fragmentation terms cause the ith moment to be coupled to the $(i+1)$th moment. This prevents us from attaining a solution for M_2, as it is coupled to M_3 which is unknown. As a result, we cannot use the weighted mass M_2/M_1 as a measure of polymer size. Instead, we focus on the mean size of polymers of size $i \geq 2$. This is given by the ratio of $\tilde{M}_1 = \sum_{i \geq 2} i n_i$ and $\tilde{M}_0 = \sum_{i \geq 2} n_i$, and for this sytem is

$$\frac{\tilde{M}_1}{\tilde{M}_0} = \frac{2-p}{1-p}, \tag{12}$$

which has a finite value in the limit $t \to \infty$ which is controlled by K, unless $k_\mathrm{f} = 0$, in which case the mean polymer size diverges linearly in time. This is illustrated in Fig. 3c.

More complex processes can be treated in a fixed-mass mean field model. For example, if monomers require activation before they can participate in polymer aggregation dynamics,

then the system is described by equations

$$\begin{aligned}\dot{n}_i &= \frac{1}{2}k_{\mathrm{p}}\sum_{j=1}^{i-1} n_j n_{i-j} - k_{\mathrm{p}} n_i \sum_{j=1}^{\infty} n_j - k_{\mathrm{f}} n_i (i-1) + 2k_{\mathrm{f}} \sum_{j=i+1}^{\infty} n_j + k_{\mathrm{A}} n_i \delta_{i,1}, \\ \dot{n}_I &= -k_{\mathrm{A}} n_I,\end{aligned} \tag{13}$$

where n_1 is the population of active monomers, n_I is the population of inactive monomers, k_{A} is the rate of activation, and the other terms are the same as before. If $k_{\mathrm{f}} = 0$, we can write an equation for the second moment M_2:

$$\dot{M}_2 = k_{\mathrm{p}}(M_1 - n_I)^2. \tag{14}$$

Regardless of the value of k_{f}, if $k_{\mathrm{A}} > 0$, the long-time behaviour is the same as the system without activation, as the inactive monomer population is depleted via activation. Instead, the effect of activation is seen at early times. For $k_{\mathrm{f}} = 0$, the first terms in a Taylor expansion of M_2 are

$$M_2 = M_2(0) + 2k_{\mathrm{p}}k_{\mathrm{A}}^2 t^3 \tag{15}$$

so that the early growth of M_2/M_1 is as t^3. This is illustrated in Fig. 3. On the other hand, if k_{f} is nonzero, we instead consider the ratio M_1/M_0, and the same Taylor expansion argument gives growth as $k_{\mathrm{p}}k_{\mathrm{A}}t^2$ [7]. It is well known, however, that the scaling of the initial dynamics depends strongly on the rules of activation [23]: (i) If no activation is necessary to form a dimer, growth is linear. (ii) If active monomers can bind to inactive monomers to form dimer, growth is quadratic. (iii) If activation of both monomer is needed to form dimers, a cubic law follows. These results are recovered by 3d simulations as shown in Fig. 1.

A second variation on the Blatz and Tobolsky model is to allow monomers to become latent, after which they no longer participate in polymerization dynamics. This is described by equations

$$\begin{aligned}\dot{n}_i &= \frac{1}{2}k_{\mathrm{p}}\sum_{j=1}^{i-1} n_j n_{i-j} - k_{\mathrm{p}} n_i \sum_{j=1}^{\infty} n_j - k_{\mathrm{f}} n_i (i-1) + 2k_{\mathrm{f}} \sum_{j=i+1}^{\infty} n_j \\ &\quad -k_{\mathrm{L}} n_i \delta_{i,1}, \\ \dot{n}_L &= k_{\mathrm{L}}(n_1),\end{aligned} \tag{16}$$

where n_L is the population of latent monomers, k_{L} is the rate of latentization, and all other terms are as before. The effect of latentization depends on whether k_{f} is nonzero, that is,

whether polymer fragmentation is allowed. If polymers can fragment, then the long-time steady state of the system is for all mass to exist as latent monomers, since these can be created but not destroyed and are therefore a 'sink' of population.

On the other hand, if $k_\mathrm{f} = 0$, then mass which is converted to polymer form remains as a polymer indefinitely and cannot latentize. In this case, we can write an equation for M_2,

$$\dot{M}_2 = k_\mathrm{p}(M_1 - n_L)^2. \tag{17}$$

At long times, the latent population $n_L \to$ const so that M_2/M_1 grows linearly in time. However, at short times, latentization causes a slowing of dynamics that depends on k_L, as illustrated in Fig. 3d.

Model with mass flux

Having seen that the Blatz and Tobolsky model of Eq. (6) is sufficient to describe long-time dynamics in systems in which the total monomer and polymer mass is conserved, we now consider the effects of allowing production and degradation of proteins. In other contexts study has been made of situations with an influx of mass [15, 24–26], annihilation reactions [13, 27, 28] and mass removal [29–33]. These studies reveal a rich variety of behaviors, and open systems can be very different to their closed counterparts.

Here, we consider a set of equations describing aggregation and fragmentation incorporating monomer production and polymer degradation. The system we study is described by

$$\begin{aligned}\dot{n}_i = \; & \frac{1}{2}k_\mathrm{p}\sum_{j=1}^{i-1} n_j n_{i-j} - k_\mathrm{p} n_i \sum_{j=1}^{\infty} n_j \\ & - k_\mathrm{f} n_i (i-1) + 2k_\mathrm{f}\sum_{j=i+1}^{\infty} n_j - k_\mathrm{out} f(i) n_i + k_\mathrm{in}\delta_{i,1}\end{aligned} \tag{18}$$

for $i \geq 1$. The difference between this system and that of Eq. (6) are the final two terms on the righthand side:

- $-k_\mathrm{out} f(i) n_i$ describes the decrease in n_i due to i-mers being removed from the system, with an i-dependent rate described by the function $f(i)$; and
- $k_\mathrm{in}\delta_{i,1}$ describes the increase in n_1 due to an influx of monomers.

A major difference between our system here and the majority of systems with mass removal studied previously is the form of the size dependence of polymer degradation. We expect that the rate of polymer degradation decreases with increasing polymer size because degradation of larger polymers is more difficult [34, 35]. The size dependence $f(i)$ is not known, so we test both exponential decay and power law decay (with exponent $\alpha \geq 1$) of $f(i)$ and find similar behavior. In previous models with mass removal, which are not intended to describe protein accumulation, the size dependence is different to the present model, for example, size independent [29, 32]; or a power law $f(i) \sim i^{-1/3}$ [33], which is more slowly decaying than the power laws we test. To our knowledge, the decay we study has not previously been studied.

We introduce a cutoff to the function $f(i)$, so that $f(i) = 0$ for $i > c$. The cutoff c is required to keep the number of equations to be solved finite. As described below, we find that for sufficiently fast decay of $f(i)$ with i, the exact value of the cutoff is not important. (With $k_{\mathrm{out}} = 0$, one can write equations for $M_0 = \sum_i n_i$ and $M_1 = \sum_i i n_i$ as described above, but this trick fails here. For example, when $c \to \infty$, a power law $f(i) \sim i^{-\alpha}$ with integer α couples the jth moment M_j to the $(j-\alpha)$th moment $M_{j-\alpha}$.) With this form for the size dependence of polymer degradation, we obtain a set of $c+2$ equations

$$\dot{\tilde{M}}_0 = \frac{1}{2}k_{\mathrm{p}} n_1^2 - \frac{1}{2}k_{\mathrm{p}} \tilde{M}_0^2 - 3k_{\mathrm{f}} \tilde{M}_0 + k_{\mathrm{f}}(M_1 - n_1) - k_{\mathrm{out}} \sum_{j=2}^{c} n_j f(j), \tag{19}$$

$$\dot{M}_1 = k_{\mathrm{in}} - k_{\mathrm{out}} \sum_{j=1}^{c} n_j j f(j), \tag{20}$$

$$\dot{n}_1 = -k_{\mathrm{p}} n_1(\tilde{M}_0 + n_1) + 2k_{\mathrm{f}} \tilde{M}_0 + k_{\mathrm{in}} - k_{\mathrm{out}} n_1, \tag{21}$$

$$\begin{aligned} \dot{n}_i = &\frac{1}{2}k_{\mathrm{p}} \sum_{j=1}^{i-1} n_j n_{i-j} - k_{\mathrm{p}} n_j(\tilde{M}_0 + n_1) \\ &- k_{\mathrm{f}} n_i (i-1) + 2k_{\mathrm{f}}(\tilde{M}_0 - \sum_{j=2}^{i} n_j) - k_{\mathrm{out}} n_i i^{-3}, 1 < i \leq c. \end{aligned} \tag{22}$$

where we have defined $\tilde{M}_0 = \sum_{i=2}^{\infty} n_i = M_0 - n_1$. We use initial conditions $n_1(0) = 1$, $n_i(0) = 0$ for all other i, and therefore $M_1(0) = 1$, $\tilde{M}_0(0) = 0$.

For simplicity, we start by fixing k_{in}, k_{p} and k_{f} and discussing a transition controlled by k_{out}. For small k_{out} (the "growing phase"), the mean polymer size grows indefinitely as t^{β}, with $\beta \to 1/2$ as $k_{\mathrm{out}} \to 0$. As k_{out} is increased (into the "stationary phase"), at long times the mean size saturates at a finite value. A typical example of this is shown in Fig. 2.

We have tested $\alpha = 1, 2, 3$ and find a sharp transition for all α values, for any finite cutoff c. However, as shown in Fig. 3, for $\alpha \leq 2$, the critical value of k_{out} has a strong dependence on c. Indeed, as $c \to \infty$, the critical point $k^*_{\text{out}} \to 0$, and the mean polymer size always reaches a stationary state. In contrast, for $\alpha > 2$, k^*_{out} has a much weaker dependence on c, and as $c \to \infty$, k^*_{out} remains finite. We have also tested a polymer degradation size dependence of $f(i) \sim \exp(-i)$, and again find a finite k^*_{out} for all c.

Allowing all four k parameters to vary, we can make an analytical argument for the location of the phase transition in the limit $c = 1$ (which is equivalent to $c > 1$ and $\alpha \to \infty$). The basis for the argument is to assume a steady state exists, and to determine what restrictions this places on parameter values.

First, note that in a steady state, we have $\dot{M}_1 = 0$, or:

$$0 = k_{\text{in}} - k_{\text{out}} n_1. \tag{23}$$

Setting $\dot{n}_1 = 0$ gives

$$0 = -k_{\text{p}} n_1 M_0 + 2k_{\text{f}}(M_0 - n_1) + k_{\text{in}} - k_{\text{out}} n_1 \tag{24}$$

and combining these two equations and rearranging gives

$$1 = \frac{1}{2} \frac{k_{\text{p}} k_{\text{in}}}{k_{\text{f}} k_{\text{out}}} + \frac{k_{\text{in}}}{k_{\text{out}}} \frac{1}{M_0}. \tag{25}$$

This relationship must hold in the steady state. Because the second term on the right hand side, $k_{\text{in}}/(k_{\text{out}} M_0)$ is never negative, if

$$\frac{k_{\text{p}} k_{\text{in}}}{k_{\text{f}} k_{\text{out}}} > 2 \tag{26}$$

then the condition (25) cannot hold and a steady state with constant mean polymer size is not possible. The condition (26) separates the parameter space into two regions. We refer to parameter values that satisfy (26) as the growing phase, and parameter values that do not satisfy (26) (i.e., those for which a non-growing steady state is not ruled out) as the stationary phase. As seen in Fig. 5, this condition serves well to predict the location of the phase transition even for $c > 1$.

Extracting effective mean-field parameters from three dimensional simulations

To extract effective mean-field parameters from three dimensional simulations, we keep track the number of polymers composed by i monomers n_i^{out} leaving the system and the

number of attachments N_p formed in a small time interval Δt. The effective degradation rate is then estimated as $\bar{k}_{\rm out} = \langle \sum_i i n_i^{\rm out}/(\Delta t \sum_i i n_i)\rangle$, where n_i is the number of polymers of size i present at that time. Similarly the effective polymerization rate is estimated as $\bar{k}_p = 2\langle N_p/(\Delta t n(n-1))\rangle$, where $n = \sum_i n_i$. The results, averaged over 10^7 time intervals and over 10 independent realizations of the simulations performed for different values of $k_{\rm out}$ and H, are reported in Fig. 7 and Fig. 8. The effective degradation rate follow a scaling law in terms of $k_{\rm out}$ and the system size H given by

$$\bar{k}_{\rm out} = \mathcal{F}(k_{\rm out} H^{1/4})/H \tag{27}$$

as shown in the inset of Fig. 7. The $1/H$ dependence of $\bar{k}_{\rm out}$ should result simply from the fact that polymers can only exit through the surface and the surface volume ratio is $1/H$. The additional non-trivial dependence in the scaling function should be due from non-trivial first-passage statistics of proteins. No simple scaling law was found for the polymerization rate, which displays a system-size dependent peak at the transition (Fig. 8). When we plot $\bar{k}_p$ as a function of $\bar{k}_{\rm out}$, however, we see that all the curves collapse below the transition, while above the transition a size-dependence is still apparent (Inset of Fig. 8).

Simulations of Amyloid-β synthesis and clearance measurements

Our simulations are based on the stable isotope labeling kinetics (SILK) protocol developed by Bateman *et al* [5, 36] to study the turnover of Aβ peptides in the brain. The experiments can be summarized as follows:

1. $^{13}C_6$-labeled leucine is infused to the bloodstream for 9 hours. Hourly measurements of labeled/unlabeled leucine in the bloodstream and cerebrospinal fluid (CSF) (measured through a lumbar catheter) indicate that the CSF labeled leucine fraction follows the same time course as that of the bloodstream. While labeled leucine is being infused, the CSF labeled/unlabeled ratio has a steady value of ~ 0.1.

2. Leucine is incorporated into Aβ peptides, which in turn forms plaques. Aβ peptides play the role of monomers in the polymerization dynamics.

3. The ratio of labeled to unlabeled Aβ in the CSF is also measured hourly for 36 hours, and is seen to increase until a plateau of ~ 0.1 is reached, before decaying.

4. The decay is approximately exponential, and the fractional clearance rate (FCR) is determined from the slope of the natural log of the labeled/unlabeled ratio plotted against time.

As discussed below, simulating this procedure requires a number of parameters. As such, the power of our simulations is not in providing a measurement of model parameters from experiments, but rather in studying the effect of varying parameters to move the system towards its critical point, and in being able to examine directly the labeled mass "in the brain" to verify that results obtained through CSF measurements are qualitatively the same as would be obtained in a direct measurement of the brain.

As discussed in the main text, our procedure for studying a change in parameters is to first run a mean field simulation using the model of Equations (19) with parameters in the stationary phase for 50 years to obtain a steady state. For simplicity, we set $c = 1$, that is, only monomers are degraded. After performing a labeling test over the course of 36 hours, as described below, we reduce the value of k_{out} slightly and continue the simulation for a further 3 years, before performing another labeling test. We repeat this several times, until the value of k_{out} is in the growing regime. We choose to vary k_{out} while fixing other parameters because in our simulations, k_{out} directly controls protein degradation. The evolution of the total mass over the course of a whole simulation is shown in Fig. 3a.

We simulate a labeling experiment as follows. During the labeling test, a fraction of monomers produced are 'labeled'. We base this fraction on the time course reported by Bateman *et al* (Fig. 3b of [5]), as shown in Supplementary Fig. 5c. labeled monomers can be incorporated into polymers, and we track the total labeled mass. During the labeling experiment, our system is described by the following equations, which can be solved numerically:

$$\dot{\tilde{M}}_0 = \frac{1}{2}k_{\text{p}}n_1^2 - \frac{1}{2}k_{\text{p}}\tilde{M}_0^2 - 3k_{\text{f}}\tilde{M}_0 + k_{\text{f}}(M_1 - n_1), \tag{28}$$

$$\dot{n}_1 = -k_{\text{p}}n_1(\tilde{M}_0 + n_1) + 2k_{\text{f}}\tilde{M}_0 + k_{\text{in}} - k_{\text{out}}n_1, \tag{29}$$

$$\dot{n}_{1L} = -k_{\text{p}}n_{1L}(\tilde{M}_0 + n_1) + 2k_{\text{f}}\tilde{M}_0\frac{\tilde{M}_{1L}}{\tilde{M}_1} + k_{\text{in}}\ell(t) - k_{\text{out}}n_{1L}, \tag{30}$$

$$\dot{M}_1 = k_{\text{in}} - k_{\text{out}}n_1, \tag{31}$$

$$\dot{M}_{1L} = k_{\text{in}}\ell(t) - k_{\text{out}}n_{1L} \tag{32}$$

where n_1 is the total number of monomers, M_1 is the total mass, n_{1L} is the total number of labeled monomers, M_{1L} is the total labeled mass, $\tilde{M}_{1L}$ is the labeled mass belonging to polymers of size $i \geq 2$, and $\ell(t)$ is the fraction of all monomers produced at time t that are labeled. (Note that this is not the same as the labeled/unlabeled ratio, because here the concentration of labeled monomers is normalized by all monomers.) We make the assumption that the probability of a labeled monomer being created by a fragmentation process is proportional to the ratio $\tilde{M}_{1L}/\tilde{M}_1$, that is, the probability a monomer fragments from a polymer does not depend on whether it is labeled.

A key issue that is not yet fully resolved is the relationship between measurements of labeled/unlabeled ratios in CSF made via a lumbar catheter and values in the brain, because flow of CSF is non-trivial [4, 37]. In our simulations, we are able to measure quantities "in the brain" directly, but we also consider the effect of mixing via a simple model [4] in which the labeled fraction in the CSF, p_{CSF} is related to the labeled fraction in the brain, p, by

$$\dot{p}_{\text{CSF}} = v(p - p_{\text{CSF}}), \tag{33}$$

where v is a mixing rate. We measure FCR using p_{CSF} measured between $t = 30\text{h}$ and $t = 36\text{h}$.

The parameters we use for the initial simulation are given in Supplementary Table I. These are selected so that the time course of the simulated CSF labeled/unlabeled ratio agrees with that reported in Fig. 3c of Ref. [5], as seen in Fig. 9. We note that our model unable to reproduce the initial 4 hour lag reported by Bateman *et al* unless we introduce a 3 hour delay in the process. It is likely the lag in the experiments is related to the complex mixing of CSF, which our model does not incorporate. We are also able to study the labeled/unlabeled ratio in the brain directly, as shown in Supplementary Fig. 5c. Comparison with experimental CSF labeled/unlabeled ratios (shown in Fig. 9) indicate that the same qualitative trends are seen.

Supplementary references

---

[1] Knowles, T. P. J., Waudby, C. A., Devlin, G. L., Cohen, S. I. A., Aguzzi, A., Vendruscolo, M., Terentjev, E. M., Welland, M. E., and Dobson, C. M. An analytical solution to the kinetics of breakable filament assembly. *Science (New York, N.Y.)* **326**(5959), 1533–7 (2009).

[2] Xue, W.-F., Hellewell, A. L., Gosal, W. S., Homans, S. W., Hewitt, E. W., and Radford, S. E. Fibril fragmentation enhances amyloid cytotoxicity. *The Journal of Biological Chemistry* **284**, 34272 (2009).

[3] Cohen, S. I. A., Linse, S., Luheshi, L. M., Hellstrand, E., White, D. A., Rajah, L., Otzen, D. E., Vendruscolo, M., Dobson, C. M., and Knowles, T. P. J. Proliferation of amyloid-β42 aggregates occurs through a secondary nucleation mechanism. *Proceedings of the National Academy of Sciences* **110**, 9758 (2013).

[4] Edland, S. D. and Galasko, D. R. Fractional synthesis and clearance rates for amyloid β. *Nature Medicine* **17**, 1178 (2011).

[5] Bateman, R. J., Munsell, L. Y., Morris, J. C., Swarm, R., Yarasheski, K. E., and Holtzman, D. M. Human amyloid-β synthesis and clearance rates as measured in cerebrospinal fluid *in vivo*. *Nature Medicine* **12**, 856 (2006).

[6] Binder, K., editor. *Monte Carlo and Molecular Dynamics Simulations in Polymer Science.* Oxford University Press, New York, Oxford, (1995).

[7] Noto, R., Santangelo, M. G., Ricagno, S., Mangione, M. R., Levantino, M., Pezzullo, M., Martorana, V., Cupane, A., Bolognesi, M., and Manno, M. The tempered polymerization of human neuroserpin. *PLoS ONE* **7**(3), e32444 (2012).

[8] Gillespie, D. T. A general method for numerically simulating the stochastic time evolution of coupled chemical reactions. *Journal of Computational Physics* **22**(4), 403 – 434 (1976).

[9] Smoluchowski, M. Versuch einer mathematischen Theorie der Koagulationskinetik kolloider Lösungen. *Z. Phys. Chem.* **92**, 215 (1917).

[10] Blatz, P. J. and Tobolsky, A. V. Note on the Kinetics of Systems Manifesting Simultaneous Polymerization-Depolymerization Phenomena. *Journal of Physical Chemistry* **49**(2), 77–80 (1945).

[11] Lushnikov, A. Evolution of coagulating systems. *Journal of Colloid and Interface Science* **54**(1), 94–101 (1976).

[12] Family, F., Meakin, P., and Deutch, J. Kinetics of Coagulation with Fragmentation: Scaling Behavior and Fluctuations. *Physical Review Letters* **57**(6), 727–730 (1986).

[13] Ben-Naim, E. and Krapivsky, P. Kinetics of aggregation-annihilation processes. *Physical Review E* **52**(6), 52 (1995).

[14] Vigil, R. D. On equilibrium solutions of aggregation-fragmentation problems. *Journal of colloid and interface science* **336**(2), 642–7 (2009).

[15] Krapivsky, P. L. and Connaughton, C. Driven Brownian coagulation of polymers. *The Journal of chemical physics* **136**(20), 204901 (2012).

[16] Jarrett, J. T. and Lansbury, P. T. Seeding "one-dimensional crystallization" of amyloid: A pathogenic mechanism in Alzheimer's disease and scrapie? *Cell* **73**(6), 1055–1058 (1993).

[17] Masel, J., Jansen, V. a., and Nowak, M. a. Quantifying the kinetic parameters of prion replication. *Biophysical chemistry* **77**(2-3), 139–52 (1999).

[18] Hall, D. and Edskes, H. Silent prions lying in wait: a two-hit model of prion/amyloid formation and infection. *Journal of molecular biology* **336**(3), 775–86 (2004).

[19] Collins, S. R., Douglass, A., Vale, R. D., and Weissman, J. S. Mechanism of prion propagation: amyloid growth occurs by monomer addition. *PLoS biology* **2**(10), e321 (2004).

[20] Kunes, K., Cox, D., and Singh, R. One-dimensional model of yeast prion aggregation. *Physical Review E* **72**(5), 051915 (2005).

[21] Xue, W.-F., Homans, S. W., and Radford, S. E. Systematic analysis of nucleation-dependent polymerization reveals new insights into the mechanism of amyloid self-assembly. *Proceedings of the National Academy of Sciences of the United States of America* **105**(26), 8926–31 (2008).

[22] Chandrasekhar, S. Stochastic problems in physics and astronomy. *Rev. Mod. Phys.* **15**, 1–89 (1943).

[23] Oosawa, F. and Asakura, S. *Thermodynamics of the Polymerization of Protein.* Academic Press, (1975).

[24] Field, G. B. and Saslaw, W. C. A Statistical Model of the Formation of Stars and Interstellar Clouds. *The Astrophysical Journal* **142**, 568 (1965).

[25] Cueille, S. and Sire, C. Droplet nucleation and Smoluchowski's equation with growth and injection of particles. *Physical Review E* **57**(1), 881–900 (1998).

[26] Ben-Naim, E. and Krapivsky, P. Condensates in driven aggregation processes. *Physical Review E* **75**(1), 011103 (2007).

[27] Krapivsky, P. L. Nonuniversality and breakdown of scaling in two-species aggregation with annihilation. *Physica A: Statistical Mechanics and its Applications* **198**(1-2), 135–149 (1993).

[28] Sillanpää, J. and Koponen, I. Island growth in ion beam assisted metal-on-metal deposition modelled by rate equations. *Nuclear Instruments and Methods in Physics Research Section B: Beam Interactions with Materials and Atoms* **142**(1-2), 67–76 (1998).

[29] Hendriks, E. and Ziff, R. Coagulation in a continuously stirred tank reactor. *Journal of Colloid and Interface Science* **105**(1), 247–256 (1985).

[30] Edwards, B., Cai, M., and Han, H. Rate equation and scaling for fragmentation with mass loss. *Physical Review A* **41**(10), 5755–5757 (1990).

[31] Singh, P. and Rodgers, G. J. Coagulation processes with mass loss. *Journal of Physics A: Mathematical and General* **29**(2), 437–450 (1996).

[32] Wattis, J. A., McCartney, D. G., and Gudmundsson, T. Coagulation equations with mass loss. *Journal of Engineering Mathematics* **49**(2), 113–131 (2004).

[33] Ben-Naim, E. and Krapivsky, P. L. Polymerization with freezing. *Journal of Physics: Condensed Matter* **17**(49), S4249–S4262 (2005).

[34] Kisselev, A. F., Akopian, T. N., and Goldberg, A. L. Range of sizes of peptide products generated during degradation of different proteins by archaeal proteasomes. *J Biol Chem* **273**(4), 1982–9 (1998).

[35] Kisselev, A. F., Akopian, T. N., Woo, K. M., and Goldberg, A. L. The sizes of peptides generated from protein by mammalian 26 and 20 s proteasomes. implications for understanding the degradative mechanism and antigen presentation. *J Biol Chem* **274**(6), 3363–71 (1999).

[36] Mawuenyega, K. G., Sigurdson, W., Ovod, V., Munsell, L., Kasten, T., Morris, J. C., Yarasheski, K. E., and Bateman, R. J. Decreased Clearance of CNS Amyloid-β in Alzheimer's Disease. *Science* **330**, 1774 (2010).

[37] Elbert, D. L., Patterson, B. W., Ercole, L., Ovod, V., Kasten, T., Mawuenyega, K., Yarasheski, K., Morris, J. C., Benzinger, T., Holtzman, D. M., and Bateman, R. J. Reply to: Fractional synthesis and clearance rates for amyloid β. *Nature Medicine* **17**, 1179 (2011).
